# Producing infectious enterovirus type 71 in a rapid strategy

**DOI:** 10.1186/1743-422X-7-116

**Published:** 2010-06-04

**Authors:** Jian-Feng Han, Rui-Yuan Cao, Xue Tian, Man Yu, E-De Qin, Cheng-Feng Qin

**Affiliations:** 1 State Key Laboratory of Pathogen and Biosecurity, Beijing Institute of Microbiology and Epidemiology, Beijing 100071, PR China

## Abstract

**Background:**

Enterovirus 71 (EV71) is an etiologic agent of hand-foot-and-mouth disease (HFMD), and recent HFMD epidemics worldwide have been associated with a severe form of brainstem encephalitis associated with pulmonary edema and high case-fatality rates. EV71 contains a positive-sense single-stranded genome RNA of approximately 7400 bp in length which encodes a polyprotein with a single open reading frame (ORF), which is flanked by untranslated regions at both the 5' and 3' ends.

**Results:**

A long distance RT-PCR assay was developed to amplify the full length genome cDNA of EV71 by using specific primes carrying a SP6 promoter. Then the *in vitro *synthesized RNA transcripts from the RT-PCR amplicons were then transfected into RD cells to produce the rescued virus. The rescued virus was further characterized by RT-PCR and indirect fluorescent-antibody (IFA) assay in comparison with the wild type virus. The rescued viruses were infectious on RD cells and neurovirulent when intracerebrally injected into suckling mice.

**Conclusions:**

Thus, we established a rapid method to produce the infectious full length cDNA of EV71 directly from RNA preparations and specific mutations can be easily engineered into the rescued enterovirus genome by this method.

## Background

Enterovirus 71 (EV71) is the major causative agent of hand, foot and mouth disease (HFMD), and may cause various neurological diseases, such as aseptic meningitis, acute flaccid paralysis and fatal encephalitis [1]. In recent years, epidemic and sporadic outbreaks of neurovirulent EV71 infections have been reported throughout the world, representing a major public health concern [2]. EV71 has become one of the most important enteroviruses known to cause fatalities in children. Therefore, understanding its virology, epidemiology, diagnosis, and prevention is of particularly importance [3].

EV71 belongs to human enterovirus species A of the genus *Enterovirus *within the family *Picornaviridae*, and it contains a single-stranded positive genome RNA of about 7,400 bp in length which is infectious when introduced into cell culture. The single open reading frame (ORF) encodes a polyprotein and is flanked by untranslated regions (UTR) at the 5' and 3' ends; a variable length poly-A tract is located at the terminus of the 3'UTR. The polyprotein can be divided into three genomic regions (P1, P2 and P3). The P1 encodes the capsid comprised of four structural proteins VP1, VP2, VP3 and VP4. The P2 and P3 encode the nonstructural proteins including 2A, 2B, 2C, 3A, 3B, 3C and 3D [4].

Reverse genetics permits the use of cDNA copies of viral RNA genomes to produce detailed studies of molecular features of virus infection and replication. The reverse genetic of picornaviruses has been developed for a long time [5]. In 1981, Racaniello and Baltimore [6] firstly demonstrated that the complete, cloned cDNA of the genome of poliovirus was infectious when transfected into permissive mammalian cells. Then, the infectious cDNA clones of coxsackievirus [7,8], hepatitis A virus [9], and so on were constructed, respectively. Different strategies have been utilized by different groups worldwide to obtain the full length cDNA of viral genome. Long distance RT-PCR technology can amplify up to 10,000 nucleotides in a rapid strategy, which has been previously described to amplify the full length cDNA of different enteroviruses [10-13]. Thus, we are particularly interested to adapt this strategy into the study of the emerging enterovirus, EV71. Here, we described a rapid strategy to amplify the full length cDNA of EV71 by long-distance RT-PCR using modified primers, and the RNA transcripts of amplicons were evidenced infectious in cell cultures and suckling mice.

## Results

**Figure 1:**
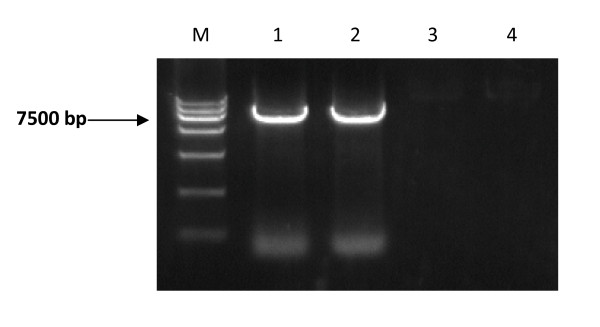
**Amplification of the full-length genomic cDNA of EV71 at different annealing temperature**. Lane 1: 50°C; lane 2: 52°C; lane 3: 55°C; lane 4: negative control; M: DL15000 Marker.

**Figure 2:**
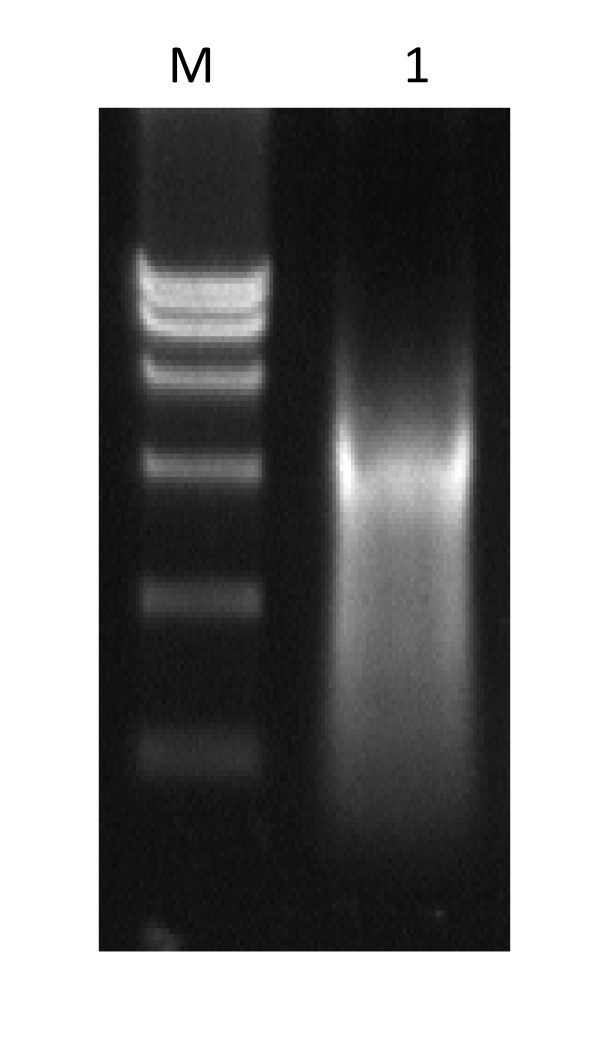
***In vitro *transcription of the full-length genomic cDNA of EV71**. Lane 1: RNA transcripts; M: DL15000 Marker.

Firstly, to amplify the full-length genomic cDNA of EV71, a long-distance RT-PCR method was established and optimized. The SP6 promoter sequence was added to upstream of the upper primer to facilitate *in vitro *transcription. After optimization of RT-PCR conditions, chiefly through turning down annealing temperature from 55°C to 52°C or 50°C, the full-length cDNA of EV71 AH08/06 strain was successfully amplified (Fig 1). Electrophoresis showed the specific single DNA product band with the expected size of about 7.5 kb which could be easily purification for further *in vitro *transcription. The cDNA amplicons were then subjected to *in vitro *transcription by the SP6 *in vitro *transcription kit (Fig 2).

**Figure 3:**
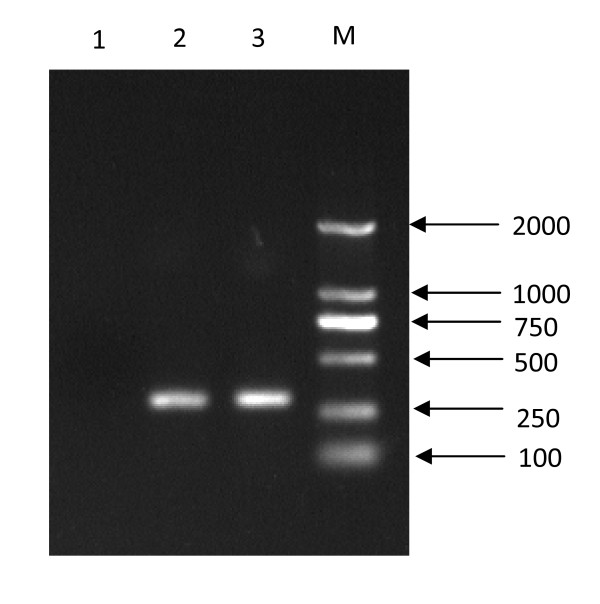
**RT-PCR identification of the rescued virus**. Lane 1: negative control; Lane 2: the rescued virus; Lane 3: wild-type virus; M: DL2000 Marker.

**Figure 4:**
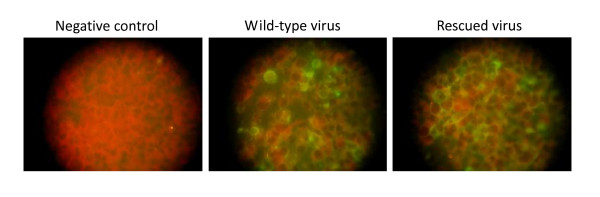
**IFA identification of the rescued virus**. EV71 VP1-specific monoclonal antibody were used according to the standard protocol. Negative and positive controls were given.

Then, the above mentioned RNA transcripts were transfected to cultured RD cells, which is sensitive to EV71. Seventy two hours post transfection, the transfected cells and supernatants were collected, and RT-PCR and IFA were performed to identify the rescued viruses, respectively. EV71-specfic RT-PCR results showed that the rescued virus had the specific band with the same size of the wile-type virus (Fig. 3), and sequencing analysis showed that it originated from the parental viruses. Further, IFA results showed that VP1 proteins can be detected in rescued virus infected RD cells and demonstrated the rescued viruses could produce viral proteins with specificity to EV71 (Fig. 4).

**Figure 5:**
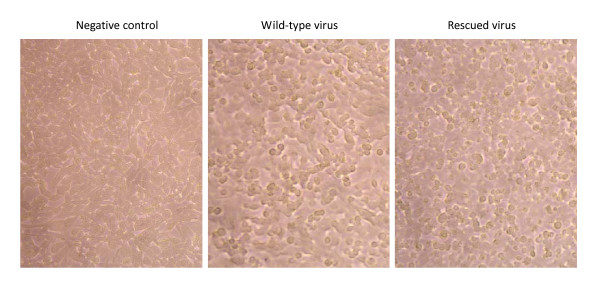
**Phenotype characteristics of the rescued EV71**. RD cells infected with the rescued and wild type EV71 were observed for CPE at 24 hrs after infection.

Further, the phenotypic properties of the rescued virus were compared with its parental virus. Similar CPE were observed 24 h post infection such as cell rounding, aggregation, fall off and floatation, etc (Fig. 5). After passage on RD cells for several generations, similar CPE phenomenon of the rescued viruses appeared repeatedly. Finally, the rescued virus were injected to one-day old suckling mice via the intracerebral route, and the mice showed typical neurological manifestation on 5 days after infection including debility, ataxia, paralysis with hind limb, etc and 20% of the infected mice finally died within 2 weeks.

## Discussion

EV71 infection commonly results in mild HFMD and sometimes associated with severe neurological diseases, such as brain stem encephalitis and poliomyelitis-like paralysis especially in children. Currently, EV71 is becoming an important public health problem, especially in developing countries [14]. In China, HFMD outbreaks caused by EV71 have been frequently reported since 2006, resulting in hundreds of deaths in children. The pathogenesis of EV71 infection is still not fully understood, and no antiviral drugs or vaccines have been approved for clinical use until now [15].

By using reverse genetics technology, specific mutations can be introduced into the full-length genomic cDNA, which will no doubt accelerate the understanding of pathogenesis and development of vaccine. Several amino acids or nucleotides mutations in EV71 genome have been identified critical for the neurovirulence of EV71 [16-19]. In these studies, the infectious clones of EV71 were constructed by ligation of two subgenomic clones, which utilized multiple RT-PCR and molecular clone steps.

Here, in this study, a convenient and efficient system to obtain the infectious full length genomic cDNA of EV71 has been established. This method deserves attention for several reasons: Firstly, full-length cDNA of EV71 can be easily obtained and sequenced by long distance RT-PCR techniques, which will forward the development of the reverse genetics of EV71 [20]. Secondly, virus preparations could be amplified directly from RNA samples, which is more safe and easy to transport or storage in comparison with the regular culture method. Thirdly, mutations can be introduced into viral genome when amplification with specific primers; here SP6 promoter was introduced into the genome successfully and other promoters such as T7 or CMV promoters could also be added. Finally, this method is applicable to other enteroviruses such as coxsackievirus, echovirus, poliovirus and so on, most of which have similar genome. Actually, long distance RT-PCR technology has been previously described to amplify full-length cDNA of several enteroviuses [10-13].

Long template amplification was usually difficult because of many factors involved in PCR [21]. To amplify the full length cDNA of EV71 genome, we designed primers about 50 nucleotides at length which is long enough for specific elongation with the 7.5 kb genome as template. For the upper primer, we added the SP6 promoter for the subsequent transcription and restriction site as a marker that discriminate the amplified cDNA sequence from the normal genomic cDNA. Besides the primer specificity, reaction conditions are also key factors for successful amplification. The experiment results suggested that lower annealing temperature (52°C or 50°C but not 55°C) was suitable for long template PCR. With specific primer pairs, optimized PCR parameters, and high fidelity enzymes, viral genome full-length cDNA were successfully amplified.

Suckling mice injected with rescued viruses partly showed typical nervous system infection manifestation and dead subsequently, which demonstrated the rescued viruses were less neurovirulent than the parental viruses. The neurovirulent difference between rescued virus and parental virus were not clear until now and need further investigation through infectious clone and *in vitro *mutation strategy [22].

In conclusion, here we described a rapid method that rescued EV71 from RNA transcripts and rescued viruses could be infectious *in vitro *and *in vivo*, whose characterization is similar with the parental viruses. This method provides an easy way to engineer the viral genome and investigate the viral properties such as mutation, virulence, and so on. This is the first in the literature to demonstrate that EV71 could be rescued through long term PCR and infectious when inoculated into suckling mice. Thus, the techniques reported here could be helpful for studies on enterovirus molecular biology and provide a useful tool for EV71 research.

## Materials and methods

### Cells and viruses

Human rhabdomyosarcoma RD cells were maintained in Dulbecco's modified Eagle's medium (DMEM, Gibco) containing 10% fetal bovine serum (FBS, ExCell) plus 2 mM L-glutamine, 100 IU of penicillin, and 100 μg of streptomycin per ml at 37°C in the presence of 5% CO_2_. EV71 strain AH08/06 was isolated from the throat swab sample of an HFMD case in 2008, China and stored at -70°C in our laboratory.

### RNA preparation and reverse transcription

Virus was cultured in RD cells and harvested by freezing and thawing for 3 times. Viral RNA was extracted from viral stock using RNeasy mini kit (QIAGEN) according to the manufacturer's instructions. The reverse transcription reaction was performed 1 hour at 42°C with Super Script II Reverse Transcriptase (Invitrogen) using RNA from viral stock as the template.

### Full-length DNA amplification

Full-length cDNAs of EV71 were acquired after long distance PCR using specific primers as follows: 5'-AGCTCC**ACGCGT**ATTTAGGTGACACTATAGGTTAAAACAGCCTGTGGGTTGCACCCACTC-3' and 5'-AGCTCC**TCTAGA**TTTTTTTTTTTTTTTTTTT TTTTTTGCTATTCTGG-3' (underlined words indicate restriction sites). Thirty five cycles of PCR amplification were carried out with the LA Taq™ Polymerase (TaKaRa) on an automated thermal cycler (Perkin Elmer). Temperatures were 94°C during denaturation (30 sec), 50°C during annealing (30 sec) and 70°C during polymerization (8 min). The final extension time was 10 min at 72°C. The DNA products were purified using QIAquick PCR Purification Kit (QIAGEN) and further quantified on a NanoDrop Spectrophotometers (Thermo Scientific).

### In vitro transcription and transfection

RNA transcription was carried out using the SP6 *in vitro *Transcription Kit (Promega) according to the manufactory's instructions. The *in vitro*-synthesized RNA transcripts were then transfected into RD cells using Lipofectamine 2000 reagent (Invitrogen). Briefly, RD cells seeded in the 24-well plates with more than 90% confluency. Lipofectamine 2000 was diluted into OptiMEM Medium (Invitrogen) and incubated for 5 min following by mixture with the RNA transcripts for another 30 min at room temperature. Then the RNA-Lipofectamine 2000 complexes (50 μl) was added directly to the plate well of containing cells and incubate at 37°C in a CO incubator. When cytopathic effect (CPE) was observed, the rescued viruses were harvested and stored at -70°C for use.

### RT-PCR

The rescued virus was identified by RT-PCR with specific primers targeted at the VP3-VP1 genes (forward primer: 5'-GCAGCCCAAAAGAACTTCAC-3', reverse primer: 5'-ATTTCAGCAGCTTGGAGTGC-3'). The reaction parameters were as follows: 95°C during denaturation (20 sec), 45°C during annealing (25 sec) and 72°C during polymerization (30 sec) within 30 cycles. The final extension time was 10 min at 72°C. The DNA products were separated using QIAquick PCR Purification Kit and further verified by DNA sequencing (Invitrogen).

### IFA

RD cells transfected with the RNA transcripts were grown on a specific slide. RD cells were washed in PBS for 3 times and incubated with mice EV71 monoclonal antibody (Chemicon) in 1:1000 dilutions for 30 min at 37°C. Then the cells were washed and incubated with fluorescein isothiocyanate (FITC)-labeled goat anti-mouse IgG (Zhongshan) diluted 1:200 in Evans Blue for another 45 min. Finally, the cells were rinsed for 30 min in PBS and visualized under a fluorescent microscope (Olympus) after dried at room temperature.

### Neurovirulence experiments

Suckling Kunming (KM) mice of 1 day old were obtained from the (Laboratory Animal Center of the Academy of Military Medical Sciences, Beijing). These newborn suckling mice were inoculated intracerebrally with 50 μl rescued viruses or RNA transcripts. The neurological manifestations and survival rate were observed and recorded for 2 weeks. The Institutional Animal Care and Use Committee approved all animal protocols.

## List of Abbreviations

EV71: enterovirus 71; HFMD: Hand, foot and mouth disease; ORF: open reading frame; UTR: untranslated regions; DMEM: Dulbecco's modified Eagle's medium; SPF: specific pathogen free; IFA: immunofluorescent assay; FITC: fluorescein isothiocyanate.

## Competing interests

The authors declare that they have no competing interests.

## Authors' contributions

JFH and RYC designed and performed the experiments and drafted the manuscript. MY and XT participated in cell culture and animal experiments. EDQ and CFQ supervised the work and edited the final version of this manuscript. All authors read and approved the final manuscript.
